# A High-Throughput Approach for Identification of Nontuberculous Mycobacteria in Drinking Water Reveals Relationship between Water Age and *Mycobacterium avium*

**DOI:** 10.1128/mBio.02354-17

**Published:** 2018-02-13

**Authors:** Sarah-Jane Haig, Nadine Kotlarz, John J. LiPuma, Lutgarde Raskin

**Affiliations:** a Department of Civil & Environmental Engineering, University of Michigan, Ann Arbor, Michigan, USA; b Department of Pediatrics and Communicable Diseases, University of Michigan Medical School, Ann Arbor, Michigan, USA; CEH-Oxford

**Keywords:** DNA extraction, drinking water, *Mycobacterium avium*, NTM, PacBio, premise plumbing

## Abstract

Nontuberculous mycobacteria (NTM) frequently detected in drinking water (DW) include species associated with human infections, as well as species rarely linked to disease. Methods for improved the recovery of NTM DNA and high-throughput identification of NTM are needed for risk assessment of NTM infection through DW exposure. In this study, different methods of recovering bacterial DNA from DW were compared, revealing that a phenol-chloroform DNA extraction method yielded two to four times as much total DNA and eight times as much NTM DNA as two commercial DNA extraction kits. This method, combined with high-throughput, single-molecule real-time sequencing of NTM *rpoB* genes, allowed the identification of NTM to the species, subspecies, and (in some cases) strain levels. This approach was applied to DW samples collected from 15 households serviced by a chloraminated distribution system, with homes located in areas representing short (<24 h) and long (>24 h) distribution system residence times. Multivariate statistical analysis revealed that greater water age (i.e., combined distribution system residence time and home plumbing stagnation time) was associated with a greater relative abundance of *Mycobacterium avium* subsp. *avium*, one of the most prevalent NTM causing infections in humans. DW from homes closer to the treatment plant (with a shorter water age) contained more diverse NTM species, including *Mycobacterium abscessus* and *Mycobacterium chelonae*. Overall, our approach allows NTM identification to the species and subspecies levels and can be used in future studies to assess the risk of waterborne infection by providing insight into the similarity between environmental and infection-associated NTM.

## IMPORTANCE

An extraction method for improved recovery of DNA from nontuberculous mycobacteria (NTM), combined with single-molecule real-time sequencing (PacBio) of NTM *rpoB* genes, was used for high-throughput characterization of NTM species and in some cases strains in drinking water (DW). The extraction procedure recovered, on average, eight times as much NTM DNA and three times as much total DNA from DW as two widely used commercial DNA extraction kits. The combined DNA extraction and sequencing approach allowed high-throughput screening of DW samples to identify NTM, revealing that the relative abundance of *Mycobacterium avium* subsp. *avium* increased with water age. Furthermore, the two-step barcoding approach developed as part of the PacBio sequencing method makes this procedure highly adaptable, allowing it to be used for other target genes and species.

## INTRODUCTION

Management of the microbial quality of drinking water (DW) is aimed primarily at minimizing illness caused by waterborne pathogens. The increasing prevalence of infections due to nontuberculous mycobacteria (NTM) (1–4), combined with growing evidence linking such infections to DW (5–10), has highlighted NTM exposure through DW as an emerging public health challenge.

Only a few of the >150 NTM species that have been described (11) cause infections in humans (10, 12, 13). Among these are NTM frequently detected in DW and DW distribution systems (DS), including species commonly associated with human infections (e.g., *Mycobacterium avium* and *Mycobacterium abscessus*) (7, 10, 13), as well as species that rarely cause infections (e.g., *Mycobacterium frederiksbergense* and *Mycobacterium aurum*) (14, 15). Molecular surveys targeting short regions of the bacterial 16S rRNA gene are widely used to profile bacterial communities in DW; however, this target does not provide sufficient resolution to distinguish NTM species from each other (13). Improved methods for NTM identification in DW are thus critical for risk assessment of NTM infection through DW exposure.

NTM detection and differentiation in environmental samples such as DW is challenging in that NTM cells are hydrophobic and relatively impermeable, rendering them difficult to lyse (16). In addition, NTM may infect and replicate within protozoa (17–19), further limiting access to their nucleic acids. Finally, sequence analysis of relatively large (>750-bp) fragments of phylogenetically informative genes, such as *rpoB* and *hsp65*, is required to differentiate NTM species, as even full-length 16S rRNA gene sequences do not provide the resolution needed for discrimination to the species and subspecies levels (13, 20, 21). While studies to date have relied on Sanger sequencing of clone libraries of such genes (22–24), this approach is labor intensive and not well suited to high-throughput screening to detect NTM in environmental samples such as DW.

A major obstacle for high-throughput differentiation of NTM species and strains is the difficulty in sequencing long DNA fragments (>750 bp) with low error rates. The high guanine-cytosine content of NTM genomes (25) provides an additional challenge by increasing the potential for positional errors in several sequencing platforms (26). van der Wielen et al. (14) and Gomez-Smith et al. (15) applied high-throughput sequencing approaches targeting *hsp65* and using the 454 and MiSeq platforms, respectively. These approaches, however, failed to differentiate between some members of the *M. avium* and *M. abscessus* complexes, were limited in differentiating NTM at the strain level, and were prone to primer cross hybridization to other bacterial species.

In this study, we sought to determine if single-molecule real-time sequencing with the PacBio platform (Pacific Biosciences, Menlo Park, CA) could overcome these obstacles. This platform achieves long read lengths (>15,000 bp) with low error rates when used in conjunction with the circular consensus sequencing (CCS) approach, making it possible to distinguish single nucleotide polymorphisms (SNPs) and allowing, in some cases, strain level differentiation (27–29). Combining an improved method of DNA recovery from NTM with PacBio sequencing, we investigated the NTM composition of DW samples from 15 households serviced by the same DW treatment plant. In doing so, we assessed the impact of water age (i.e., combined residence time in the DS and stagnation time in premise plumbing) on the relative abundance of NTM species.

## RESULTS

### Evaluation of DNA extraction methods.

**FIG 1:**
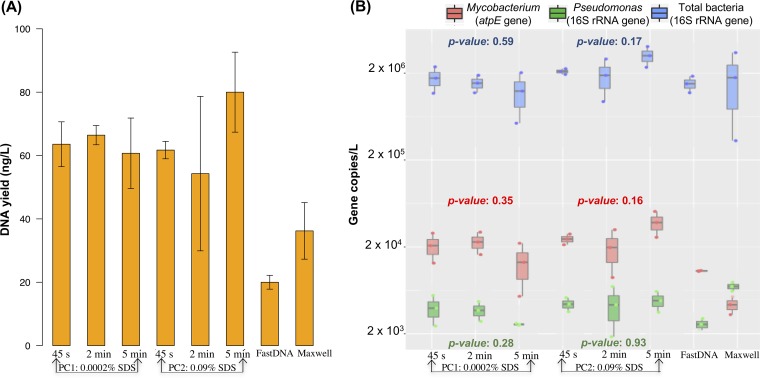
DNA extraction yields (A) and concentrations of NTM, *Pseudomonas*, and total bacteria as determined by qPCR targeting the NTM *atpE*, *Pseudomonas* 16S rRNA, and bacterial 16S rRNA genes, respectively (B), for four different DNA extraction methods on biomass filtered from 1 liter of DW. Bars and points represent the average values of triplicate experiments with the SD represented by whiskers. PC1 and PC2 are phenol-chloroform methods using the modified UNEX buffer (Table 1) with bead-beating times of 45 s, 2 min, and 5 min. *P* values represent the statistical significance of differences between bead-beating times within each method for each microbial group. FastDNA is the MP Biomedicals FastSpin kit for soil, and Maxwell is the Promega Maxwell LEV Blood DNA kit.

Two phenol-chloroform methods (designated PC1 and PC2) and two commercially available DNA extraction spin kits (FastDNA and Maxwell) (Table 1) were compared with respect to their total and NTM DNA yields from DW biomass. The phenol-chloroform-based methods yielded two to four times as much total DNA as the kits and significantly more NTM DNA (*P* < 0.001), as determined by measuring NTM abundances by quantitative PCR (qPCR) targeting the *atpE* gene (Fig. 1). However, the *Pseudomonas* DNA yields of the four extraction methods, as measured by a qPCR assay targeting the 16S rRNA gene, were comparable (Fig. 1B). PC2 employed a higher sodium dodecyl sulfate (SDS) concentration than PC1 and, when used with 5 min of bead beating, resulted in significantly greater total DNA yields (*P* = 0.043) and NTM abundances (*P* = 0.045) (Fig. 1B) than the other conditions for PC2 and PC1. There were no significant differences in the total DNA yields obtained with increased bead-beating times (from 45 s to 2 min to 5 min) by either PC1 or PC2 (Fig. 1A) (*P* = 0.10, 0.19, and 0.66, respectively). Likewise, there was no significant difference in the total bacterial, NTM, and *Pseudomonas* abundances obtained with increased bead-beating times (from 45 s to 2 min to 5 min) by either PC1 or PC2 (Fig. 1B).

**TABLE 1:** Summary of the DNA extraction methods used in this study

Method	Abbreviation	Procedure
FastDNA spin kit for soil^a^	FastDNA	Mechanical (bead beating) and chemical lysis; genomic DNA purified via solutions in kit with spin filter columns
Maxwell LEV Blood DNA kit^b^	Maxwell	As described by Webster et al. (53); mechanical (bead beating) and chemical lysis; genomic DNA purified via solutions and magnetic beads in kit
Phenol-chloroform DNA extraction	PC1	Chemical lysis with modified UNEX buffer^c^ with 0.0002% SDS and mechanical lysis (bead beating) for 45 s or 2 or 5 min
Phenol-chloroform DNA extraction	PC2	Chemical lysis with modified UNEX buffer with 0.09% SDS and mechanical lysis (bead beating) for 45 s or 2 or 5 min

a MP Biomedicals, Solon, OH.

b Promega, Madison, WI.

c Modified UNEX buffer composition (final concentrations): 2.22 M guanidinium thiocyanate (MP Biomedicals, Solon, OH), 0.0042% Tween 20, 0.0691 M sodium acetate, 0.1185 M sodium chloride, 0.1975% sodium sulfite, 0.0988% dithioerythritol, and 816 milliAnson U (1 milliAnson U is defined as the amount of enzyme that liberates Folin-positive amino acids and peptides corresponding to 1 μmol of tyrosine under assay conditions in 1 min with hemoglobin as the substrate) of proteinase K (Ambion, Waltham, MA).

As PC2 with 0.09% SDS and 5 min of bead beating yielded the greatest total DNA and NTM abundance, it was selected as the extraction method for the remainder of this study. The extraction efficiency of this procedure was determined by spiking 1 liter of phosphate-buffered saline (PBS) with *Escherichia coli* or *M. abscessus* cells. By using extraction-independent fluorescence microscopy to enumerate *E. coli* and *M. abscessus* cells and assuming a theoretical DNA content per cell, the theoretical DNA yield was estimated and compared with the measured DNA yields, revealing extraction efficiencies of 78.6% ± 9.5% and 26.7% ± 6.3% for *E. coli* and *M. abscessus* cells, respectively (see Text S1 in the supplemental material).

10.1128/mBio.02354-17.1 TEXT S1  Extraction efficiency calculation. Download TEXT S1, PDF file, 0.04 MB. Copyright © 2018 Haig et al. 2018 Haig et al. This content is distributed under the terms of the Creative Commons Attribution 4.0 International license.

### Evaluation of NTM identification method.

**FIG 2:**
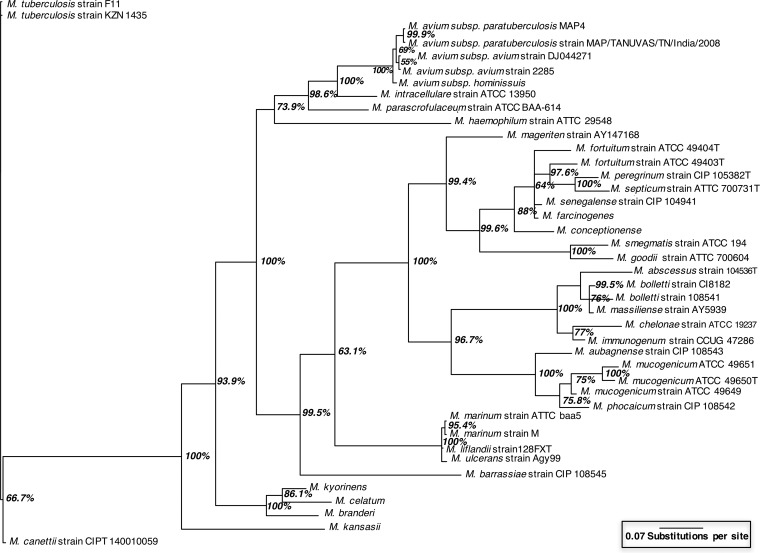
Phylogenetic tree created from sequence analysis of the 942- to 957-bp targeted section of the *rpoB* gene from 41 NTM for which full-length *rpoB* sequences are available in the GenBank database. The percentage on each branch indicates the Bayesian posterior probability value, and branch length indicates the relative inferred evolutionary distance between isolates. An *rpoB* gene sequence from *Mycobacterium tuberculosis* was used as the outgroup.

Newly developed *rpoB* primers (see Text S2) produced amplicons of various lengths (942 to 957 bp) and enabled the differentiation of the 41 NTM that currently have full-length *rpoB* sequences in the GenBank database, including species and subspecies within the difficult-to-distinguish *M. avium* complex (*M. avium*, *M. avium* subsp. *paratuberculosis*, and *Mycobacterium intracellulare*) and subspecies of *Mycobacterium fortuitum* and *Mycobacterium mucogenicum* (Fig. 2). Over the *rpoB* amplicon, there were 3 to 125 variable regions distinguishing NTM, representing SNPs and insertion sites.

10.1128/mBio.02354-17.2 TEXT S2  *rpoB* and PacBio primer and thermocycling conditions used in this study. Download TEXT S2, PDF file, 0.1 MB. Copyright © 2018 Haig et al. 2018 Haig et al. This content is distributed under the terms of the Creative Commons Attribution 4.0 International license.

**FIG 3:**
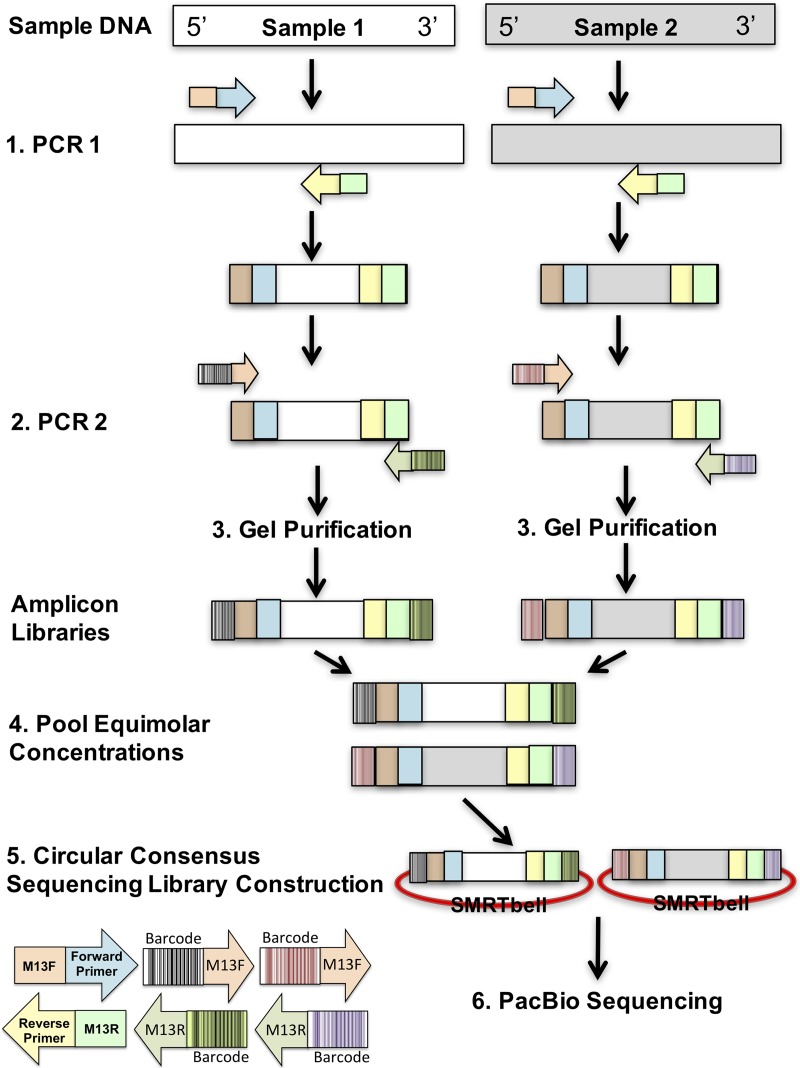
Schematic overview of the PacBio barcoding approach. For simplicity, this schematic shows how amplicon libraries are created from two separate samples. The approach includes two PCRs, the first specific to the target with M13 motifs on the 5′ and 3′ ends of the forward and reverse primers, respectively. The second PCR uses the product from step 1 and involves the addition of unique forward and reverse barcodes to the amplicons. Overall, the amplicon library created for each sample possesses a unique combination of barcodes, allowing both sample libraries to be pooled and distinguished postsequencing. Upon pooling, equal concentrations of the amplicon libraries (step 4) are submitted for PacBio sequencing by the CCS approach entailing circularization of the amplicons (SMRTbell).

**FIG 4:**
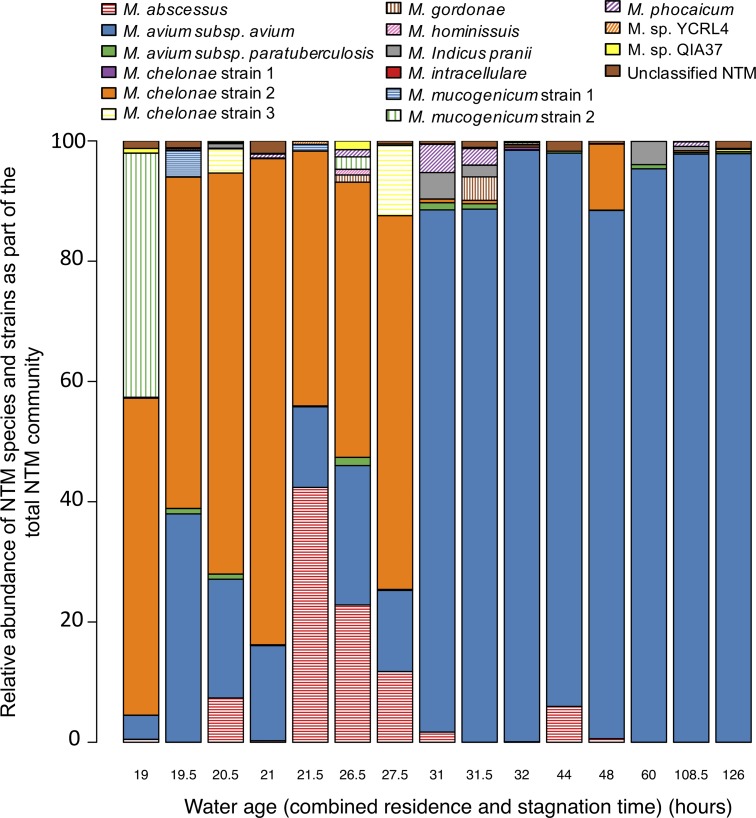
Relative abundances of NTM species and strains as part of the total NTM community in biomass from 15 DW samples collected from premise plumbing. Water age represents the total of water residence time in the distribution system and stagnation time in premise plumbing.

In multiplex sequencing, reads are sorted into sample libraries on the basis of the detection of a unique, sample-specific barcode added to the DNA to be sequenced. We implemented a two-step PCR approach in which *rpoB*-targeted primers containing an M13 motif amplify the template in the first step. A dilution of the amplicons produced by this step served as the template for successive amplification with bar-coded primers containing complements of the M13 motifs (Fig. 3). To evaluate whether our *rpoB* primers and barcoding strategy could be used for NTM differentiation in DW, *rpoB* amplicons from 16 DW samples were pooled. Fifteen of the samples consisted of biomass from 1 liter of DW collected from homes after at least 6 h of stagnation in premise plumbing, and the 16th sample was a positive control. After quality filtering, 49,728 sequences remained and >99% of them were taxonomically classified as *Mycobacterium* at a sequence identity of ≥85% (30). NTM species and strains were assigned at a sequence identity of ≥99.8%. The *rpoB* amplicons from the positive control, a mixture of two NTM operational taxonomic units (OTUs), were correctly identified as *M. avium* subsp. *avium* and *M. chelonae* (Fig. 4). The experimentally determined abundances of these two OTUs were very similar (97.5 and 2.5%) to those expected (95 and 5%, respectively). The taxonomic and abundance matches obtained for this positive control suggested a high level of confidence for NTM identification in DW samples by this sequencing method.

### NTM identification in DW.

DW samples were collected from 15 homes serviced by the same municipal DW treatment plant (Ann Arbor, MI). The DS water residence times of these homes varied from 11 to 113 h according to the city’s hydraulic model. Homes were divided into groups near (<24-h DS residence time) and far from (>24-h DS residence time) the DW treatment plant, corresponding to low and high water ages (i.e., DS residence time combined with premise plumbing stagnation time), respectively.

The sequencing effort resulted in 3,059 ± 1,023 (average ± standard deviation [SD]) NTM *rpoB* gene sequences per sample. The number of NTM OTUs present in DW samples ranged from 4 to 10 (see Text S3). Overall, 16 NTM OTUs were identified, including three unclassified and nine classified NTM species, representing both rapidly (e.g., *M. abscessus*) and slowly (e.g., *M. avium*) growing species and including three distinct strains of *M. chelonae* and two strains of *M. mucogenicum* (Fig. 4). There was no statistically significant difference in NTM diversity between the water age groups (*P* = 0.057), as measured by using the Shannon index (see Text S3). DW samples with a lower water age had a more even NTM community structure (average Pielou’s evenness ± SD of 0.49 ± 0.11), and *M. avium* subsp. *avium*, *M. abscessus*, and several *M. chelonae* strains comprised the majority of the NTM community. In comparison, DW samples with a higher water age exhibited a more uneven NTM community structure (average Pielou’s evenness of 0.17 ± 0.09) (Fig. 4). *M. avium* subsp. *avium* dominated samples with a higher water age.

10.1128/mBio.02354-17.3 TEXT S3  Combined qPCR and PacBio data. Download TEXT S3, PDF file, 0.05 MB. Copyright © 2018 Haig et al. 2018 Haig et al. This content is distributed under the terms of the Creative Commons Attribution 4.0 International license.

Permutational multivariate analysis of variance revealed that the NTM community structures of the two water age groups were significantly different from each other (*P* = 0.001), with water age explaining 81% of the difference (*P* = 0.03). The residual monochloramine concentration (*C*) multiplied by water age (i.e., the combined DS residence time and premise plumbing stagnation time [*t*]), referred to collectively as *Ct*, was not a significant parameter in the multivariate model explaining NTM community structure. However, a greater *Ct* was significantly associated with a greater NTM qPCR abundance (*P* < 0.001), with the average concentration across all samples corresponding to 6.47 × 10^6^ ± 1.03 × 10^6^ NTM *atpE* gene copies/liter. Further, additional multivariate analysis using estimated *M. avium* subsp. *avium* concentrations, obtained by combining *M. avium* subsp. *avium* relative abundances determined by the PacBio sequencing method with NTM qPCR concentrations (see Text S4), revealed that neither the monochloramine concentration nor *Ct* was a significant factor explaining *M. avium* subsp. *avium* abundance (*P* = 0.90 and 0.08, respectively).

10.1128/mBio.02354-17.4 TEXT S4  Concentrations (number of cells per liter) of NTM species and strains in biomass from 15 DW samples collected from premise plumbing. Concentrations were calculated by combining qPCR and PacBio data. Water age represents the total of water residence time in the DS and the stagnation time in premise plumbing. Download TEXT S4, PDF file, 0.7 MB. Copyright © 2018 Haig et al. 2018 Haig et al. This content is distributed under the terms of the Creative Commons Attribution 4.0 International license.

## DISCUSSION

### Improved NTM DNA recovery.

DNA extraction methods impact the abundance and types of microorganisms recovered from complex environmental and biological samples owing to physiological differences among microorganisms and differing extraction efficiencies (31, 32). Given the health risks posed by certain NTM species that can be found in DW, high-throughput methods for NTM identification in DW are needed.

Previous studies assessing the abundance of NTM in DW (14, 22, 23, 33) have generally relied on commercially available DNA extraction kits. These kits typically incorporate only modest physical disruption of bacterial cells (e.g., bead-beating times of <2 min) and do not include reagents necessary for efficient nucleic acid extraction from a range of hard-to-lyse microorganisms (34–36). Our study revealed that two commonly used commercial DNA extraction kits performed poorly in comparison with a phenol-chloroform procedure (PC2) that recovered, on average, eight times as much NTM and three times as much total DNA from DW biomass. The better performance of the PC2 method is attributed to more aggressive physical, enzymatic, and chemical lysis of NTM cell walls, which consist of peptidoglycan, arabinogalactan, and mycolic acids that are covalently linked to form a hydrophobic, relatively impermeable complex (16, 37). Specifically, the longer bead-beating time and increased concentrations of proteinase K and surfactants (SDS and Tween 20) likely improved physical lysis and aided in solubilizing and denaturing proteins (38) in the NTM cell wall, respectively. However, although PC2 yielded significantly more total DNA than the commercial kits, using this method did not result in significant differences in the abundance of bacterial 16S rRNA genes. This apparent discrepancy may be explained by differences in the potential of the extraction methods used to obtain other fractions of DNA, including DNA of *Eukarya*, which have complex cell walls, making them difficult to lyse (35).

Despite the impact DNA extraction procedures have on the recovery of different bacterial groups, few studies report DNA extraction efficiencies, making comparisons between studies challenging. In this study, we found that PC2 resulted in an extraction efficiency of 26.7% for NTM, which was three to eight times as high as the extraction efficiencies achieved with the commercial kits tested. While this is significantly lower than the extraction efficiency of approximately 45% reported by Kaevska and Slana (39), the lack of an extraction-independent method to verify the concentrations of cells used to spike samples limits the interpretation of their results. In contrast, we computed our extraction efficiency by comparing the measured DNA yields (see Text S1) to a theoretical DNA yield determined by enumeration of cells by an extraction-independent method. Conversely, recovery of Gram-negative bacteria (*E. coli*) by the PC2 method was 78.6%, considerably higher than values reported in another study, in which the extraction efficiency did not exceed 43.3% (40). Collectively, our results indicate differences in extraction efficiency between commercially available kits and our phenol-chloroform method using a custom lysis buffer with respect to NTM in DW samples. Future studies focused on risk assessment or accurate quantification of NTM should include positive DNA extraction controls to assess method effectiveness. Without such controls, results cannot be put into context and artificially low extraction yields will result in underestimation of the levels of NTM in DW (31).

### Water age and NTM distribution.

The development of *rpoB* primers (see Text S2) combined with a high-throughput PacBio sequencing assay (Fig. 3) allowed us to determine the diversity of NTM in DW samples without the need for cultivation. Furthermore, the two-step PCR and barcoding approach reduced template DNA requirements, which is useful for samples that have low biomass concentrations, such as DW samples. In addition, the barcodes with M13 motifs can be used with other gene targets, thereby removing the need to purchase gene-specific, individually barcoded primers for different assays.

Unlike previous studies employing high-throughput screening methods for NTM in environmental samples (14, 15), this study achieved species, subspecies, and in some cases strain level resolution because of the superior differential power of the *rpoB* gene (Fig. 2). Optimization of the primer set resulted in the amplification of significantly fewer non-NTM sequences (0.06% in this study compared to 68% in the study of van der Wielen et al. [14], which used the *hsp65* gene). Hence, fewer sequences needed to be discarded, making this new methodology more economical and effective. Application of the sequencing approach to DW samples revealed 13 NTM species, 5 of which are considered clinically relevant (10, 12, 13), including *M. abscessus*, *M. avium* subsp. *avium*, *M. chelonae*, *M. intracellulare*, and *M. mucogenicum*, and have been identified by previous culture-based DW studies (7, 8, 41, 42). The striking difference between the NTM present in DW reported in this study, including several clinically relevant NTM species, and the NTM present in Dutch DW (14), consisting mainly of uncultured NTM with unknown clinical relevance, calls for future research. It is possible that this difference is due to the differing disinfection regimes employed: chloramination with an average concentration of 2.04 mg/liter as Cl_2_ in this study versus the absence of a residual disinfectant in Dutch DW DS. NTM are resistant to chlorine and chloramines (42); thus, chloramine likely exerted a selective pressure in the DS in this study. Conversely, the diversity of NTM species observed in DW samples in this study was similar to that found in homes in the Netherlands (14). However, the NTM diversity was 50% lower than that found in DS (14) and biofilm samples collected from water mains in St. Paul, MN, which also contained chloramine as the residual disinfectant, albeit at a higher concentration (an average of 3.5 mg/liter as Cl_2_) than in this study (15). Such differences in NTM diversity are plausible because of the differing sample types (planktonic biomass versus biofilm), source water quality across geographic locations (Michigan versus the Netherlands versus Minnesota), disinfection practices (residual disinfection with chloramine versus no residual disinfection), and DNA extraction methods used (phenol-chloroform versus the MP Biomedical FastSpin kit for soil).

Previously, an increase in NTM abundance with water age was observed in a simulated DW DS (33); however, differences in NTM species and strain distribution for different water ages have not been described. Such differences in NTM community evenness and membership for different water ages may be due to variable growth rates and susceptibility of NTM to disinfectants and their by-products (43, 44), although more work is needed to identify the specific selective pressures that impact NTM profiles in DW. In addition, it was surprising that the NTM community compositions at relatively similar water ages of 27.5 and 31 h were dramatically different (Fig. 4). This may be due to inaccurate estimates of DS residence times, which were calculated by using the city’s hydraulic water distribution model calibrated by using average water demand values across 2010 (winter 2015-2016 water demand values, corresponding to the sampling period, were not available). More accurate DS residence times could be obtained by improving the hydraulic water distribution model and calibration, but that was beyond the scope of this study.

Combining relative abundances of NTM determined by the PacBio sequencing method with NTM qPCR concentrations (see Text S3 and S4) suggests that the concentrations of potentially clinically relevant NTM in DW samples (e.g., 3.53 × 10^4^ ± 3.32 × 10^4^ *M. avium* subsp. *paratuberculosis atpE* gene copies/liter) may have been sufficient to result in NTM infections, as previously demonstrated through dose-response models (N50 [the dose at which 50% of the population is expected to be affected], 2.70 × 10^2^ CFU) (45). Since these results were obtained with DNA-based methods that include DNA associated with nonviable NTM, it will be important in future work to validate the results obtained by methods that separate viable NTM (e.g., culture-independent methods that allow separation of viable cells [46]) prior to sequencing efforts to determine the risk of NTM infection posed by DW exposure. Furthermore, before infection mitigation strategies can be developed, future research must focus on determining the virulence of the potentially clinically relevant DW strains. This is of particular importance, as previous work has shown that environmentally isolated opportunistic pathogens can be as virulent as disease-causing, clinically isolated strains (47).

### Conclusion.

Methods are needed that yield high NTM DNA levels during extraction from low-biomass samples and recover relatively large, phylogenetically informative segments of NTM DNA to advance our understanding of the potential health risks posed by NTM in DW systems. Our use of a DNA extraction method that employs enhanced physical disruption and chemical lysis of NTM cells resulted in considerably greater yields of NTM DNA from DW samples than those obtained with commonly used commercial DNA extraction kits. The development of a two-step barcoding procedure with PacBio sequencing provided a high-throughput method to detect and differentiate NTM present in DW. Applying these methods to DW samples collected from homes receiving municipally treated DW suggests that water age impacts NTM distribution in DW. Most notably, our findings indicate that DW from homes with DS residence times of >24 h had higher concentrations of *M. avium* subsp. *avium* than DW from homes closer to the treatment plant. If supported by future, larger-scale studies, the factors contributing to this intriguing finding can be elucidated. This will, in turn, allow for better estimates of the health risks posed by NTM in DW and will provide a foundation for studies to mitigate this risk.

## MATERIALS AND METHODS

### Water sample collection and monochloramine analysis.

From October 2015 to February 2016, 1-liter cold water samples were collected from kitchen faucets after at least 6 h of stagnation from 15 homes serviced by the Ann Arbor, MI, DW treatment plant. This first 1-liter sample represents the cold water in each home’s plumbing; the minimum premise plumbing volume for the homes sampled was 3.5 liters. The DW treatment plant obtains raw water from the Huron River (80 to 85%) and from groundwater wells (15 to 20%) and provides lime softening, coagulation, flocculation, sedimentation, ozonation, filtration, and chloramination (48). The homes were located in two broadly defined areas, (i) close to the treatment plant, receiving water with shorter residence times in the DS (<24 h), and (ii) farther from the plant, receiving water with longer residence times (>24 h).

The temperature, pH, and total and free chlorine concentrations of all samples were measured on site. The average (±SD) water temperature and pH of the 15 samples were 20.4°C ± 1.9°C and 8.6 ± 0.6, respectively. Total and free chlorine concentrations were measured with a DR900 spectrophotometer (Hach, Loveland, CO). The combined chlorine (monochloramine) concentration was estimated by subtracting the free chlorine concentration from the total chlorine concentration. DS residence times for each home sampled were estimated by performing 168 h (7 days) of simulations in EPANET (49) for each home by using the City of Ann Arbor’s hydraulic water distribution model and average daily water demands across 2010 for each home. The monochloramine concentration (*C*) multiplied by water age (i.e., the combined DS residence time and premise plumbing stagnation time [*t*]), referred to collectively as *Ct*, was calculated for each sample.

Biomass was filtered from each sample with a 0.2-μm polycarbonate filter (Isopore Membrane Filters; EMD Millipore, Billerica, MA) and stored at −80°C.

### DNA extraction methods.

Four DNA extraction methods were tested in triplicate with 1-liter samples of DW from a composite sample of 30 liters of cold water collected from the Environmental and Water Resources Engineering Building at the University of Michigan (Ann Arbor). The four methods tested included the commercially available FastSpin kit for soil (MP Biomedicals, Solon, OH) and Maxwell LEV Blood DNA kit (Promega, Madison, WI) and two variations of a standard phenol-chloroform method (PC1 and PC2) with a modified version of the universal nucleic acid extraction buffer (35) (Table 1). The two phenol-chloroform-based methods (PC1 and PC2) differed in surfactant concentration. Details of the procedure are provided in Text S5. DNA extractions with the FastSpin and Maxwell kits were performed as outlined by Haig et al. (50) and Webster et al. (51), respectively. The performance of the different extraction procedures was evaluated by qPCRs targeting NTM, *Pseudomonas*, and total bacteria as described below. Subsequently, DNA was extracted from 15 DW samples collected from homes and one mock community by PC2, the phenol-chloroform-based extraction method with 0.09% SDS and 5 min of bead beating.

10.1128/mBio.02354-17.5 TEXT S5  Detailed extraction procedure used in this study. Download TEXT S5, PDF file, 0.1 MB. Copyright © 2018 Haig et al. 2018 Haig et al. This content is distributed under the terms of the Creative Commons Attribution 4.0 International license.

### Extraction efficiency.

The extraction efficiency of method PC2 was determined by spiking 1 liter of PBS with *E. coli* ATCC 15597 at 1.6 × 10^7^ ± 7.1 × 10^6^ CFU/liter or *M. abscessus* ATCC 19977 at 1.4 × 10^7^ ± 1.1 × 10^7^ CFU/liter. The cultures were grown in accordance with ATCC standard procedures. Resulting DNA yields were measured with the Qubit Fluorometer (Life Technologies, Inc., Waltham, MA). Final extraction efficiencies were calculated by comparing the measured results to the extraction-independent total cell counts determined by staining with 4′,6-diamidino-2-phenylindole (DAPI; Thermo, Fisher Scientific, Waltham, MA) and visualization with a Zeiss Axio Observer D1 fluorescence microscope (Zeiss, Peabody, MA). Theoretical DNA yields from *E. coli* and *M. abscessus* cells were calculated as outlined by Dolezel et al. (52) (see Text S3).

### qPCR.

qPCR assays targeting genes for NTM, *Pseudomonas*, and total bacteria were performed by previously published methods (34, 53, 54) as detailed in Text S6. All DNA samples were processed in triplicate with negative controls and standards. Assays were conducted with 96-well polypropylene plates on a CFX96 real-time quantitative thermocycler (Bio-Rad, Hercules, CA). Each 10-μl reaction mixture contained 5 μl of Fast EvaGreen qPCR master mix (Biotium, Fremont, CA), 0.8 μl of each primer (0.4 μM; IDT, Coralville, IA), 2.4 μl of water, and 1 μl of template DNA (~0.2 ng ⋅ μl^−1^). PCR conditions for NTM and total 16S rRNA gene assays consisted of 40 cycles, whereas the *Pseudomonas* assay was performed with 35 cycles. Melting curve analysis of the PCR products was conducted following each assay to confirm that the fluorescence signal originated from specific PCR products and not from primer dimers or other artifacts. For all qPCR assays, a linear relationship between the log of the standards’ DNA copy number and the calculated threshold cycle value across the specified concentration range was confirmed (*R*^2^ value of >0.99 in all cases). Amplification efficiencies, calculated by the method described by Pfaffl (55), varied from 1.8 to 2.0 across all assays, and these values are consistent with those reported in other studies (54, 56).

10.1128/mBio.02354-17.6 TEXT S6  qPCR primers and thermocycling conditions used in this study. Download TEXT S6, PDF file, 0.1 MB. Copyright © 2018 Haig et al. 2018 Haig et al. This content is distributed under the terms of the Creative Commons Attribution 4.0 International license.

### *rpoB* primer design.

The 41 currently available full-length NTM *rpoB* DNA sequences in the GenBank database (57) were aligned with Kalign (58) and included several strains of *M. avium* subsp. *avium*, *Mycobacterium bolletii*, *M. chelonae*, *Mycobacterium marinum*, and *M. mucogenicum*. The primers developed by Macheras et al. (59) were modified on the basis of this alignment, resulting in amplicons of 942 to 957 bp (see Text S2). Subsequent *in-silico* analysis suggested that certain versions of the degenerate primers would hybridize with non-NTM species. Therefore, equimolar concentrations of individual, nondegenerate versions of the primers that primarily amplified NTM were combined to improve primer specificity. The nondegenerate forward and reverse primer stocks comprised six forward and four reverse primers, respectively (see Text S2).

MrBayes version 3.2 (60) was used to create a Bayesian inference phylogenetic tree with alignment performed with ClustalW. Specifically, the 942- to 957-bp amplicons were fitted to a general time-reversible gamma-distributed rate variation model by using a Markov chain Monte Carlo analysis performed for 10^6^ generations with sampling every 10^3^ generations. The average SD of split frequencies was <0.007 after 10^6^ generations, indicating convergence. Posterior probabilities were averaged over the final 75% of trees (25% burn-in). A phylogenetic tree was plotted with FigTree v1.4.2.

### PacBio RS II sequencing.

Identification of NTM to the species level was performed with 15 DW samples collected in Ann Arbor, MI, and a control sample consisting of 95% *M. avium* subsp. *avium* (DJO-44271) and 5% *M. chelonae* (19237) by a two-step PCR procedure similar to that described in reference 61 (Fig. 3). The control sample was generated by combining the aforementioned two NTM species at the ratio stated after individually amplifying pure cultures of them. In the first PCR, the NTM *rpoB* genes were amplified with the newly designed *rpoB* primers (see Text S2) with either M13F or M13R motifs amended to the 5′ ends of the primers (Fig. 3). In the second PCR, products from the first PCR were amplified with primers that consisted of M13F or M13R motifs at the 3′ end and unique 16-bp PacBio barcodes at the 5′ end (see Text S2). The resulting amplicons were composed of asymmetrical barcodes (Fig. 3). Each 10-μl reaction mixture contained 1× Phusion Flash High-Fidelity PCR master mix (Thermo, Fisher Scientific, Waltham, MA), 0.4 μM each primer (IDT, Coralville, IA), 0.625 mg/ml bovine serum albumin (Life Technologies, Inc., Waltham, MA), 0.5 M betaine, 5% dimethyl sulfoxide (Sigma-Aldrich, St. Louis, MO), and 1 μl of template. The thermal cycling conditions used for each PCR are shown in Text S2. To reduce chimera formation and PCR biases, cycle numbers were kept as low as possible. The second PCR was performed 9 to 18 separate times with products purified with the Qiagen Gel Cleanup kit (Qiagen, Germantown, MD) and precipitated with PEG 6000 (50), and the resulting yields were measured with the Qubit Fluorometer (Life Technologies, Inc., Waltham, MA). Equimolar concentrations of each sample (15 ng/μl) were pooled. Amplicons were processed without shearing, and SMRTbell libraries were constructed in accordance with the PacBio 1-kb Template Preparation protocol for CCS, allowing a single insert to be sequenced multiple times (29). The polymerase-template complexes were immobilized at the bottom of the zero-mode waveguides with megabead loading and sequenced on a PacBio RS II system with C2 sequencing chemistry and one SMRT cell (University of Michigan, Ann Arbor, MI). A 1-kb collection protocol was utilized with a 180-min movie window for signal detection.

PacBio data processing and analysis. PacBio data processing was performed by the Bioinformatics Core at the University of Michigan and consisted of demultiplexing CCS by the RS_ReadsOfInsert protocol on the PacBio SMRTanalysis Portal with the following parameters: a full-pass number of >10, a predictive accuracy of >90%, and the most flexible barcode stringency.

Phylogeny of OTUs in the noncontrol samples was established on the basis of parameters obtained by testing a UPARSE analysis workflow (62) with the control sample. The resulting workflow consisted of (i) employing CUTADAPT (63) with forward and reverse M13 sequences to delineate full amplicon sequences, (ii) using USEARCH (64) to discard reads with >1.0 total expected errors, (iii) removing equipment- and reagent-derived DNA (background contamination) from dereplicated reads by using the neutral-model-based approach developed by Bassis et al. (65), (iv) clustering into OTUs with a minimum size of two reads, and (v) employing the UTAX algorithm to assign lineage information to those OTUs on the basis of a custom-made *rpoB* database. For OTUs that could not be assigned to a species/strain or were assigned with a low score (confidence value of <0.8), we employed BLAST (66) to identify the closest relatives.

The custom-made *rpoB* database was generated by downloading all 70,507 currently available bacterial *rpoB* sequences from GenBank (57), identifying the relevant amplicon sequences with USEARCH and the primer pair sequences (allowing an edit distance of 4 for each primer), and retrieving lineage information from the NCBI taxonomy database (67). Five yeast RPB2 sequences were used as decoys to train the UTAX algorithm on the bacterial data set. The resulting database consisted of 11,572 *rpoB* sequences. By using the aforementioned workflow, classifications were assigned to OTUs from the experimental samples by using global alignments with USEARCH and an identity threshold of >99.8%.

### Statistical analyses.

Significant differences between the total DNA yields and *Mycobacterium*, *Pseudomonas*, and total bacterial gene copy numbers obtained by different extraction methods were identified by Wilcoxon tests. Significant differences in the NTM community structure were determined by nonparametric multiple analysis of variance. All statistical analyses were performed with the R statistical software (68), with statistical significance determined by *P* values of <0.05.
