# Supplementary material for: A High-Throughput Approach for Identification of Nontuberculous Mycobacteria in Drinking Water Reveals Relationship between Water Age and Mycobacterium avium
Source: mBio. 2018 Feb 13;9(1):e02354-17. doi: 10.1128/mBio.02354-17 (PMC5821076; doi:10.1128/mBio.02354-17)
Supplement: TEXT S1 [file mbo001183725s1.pdf]

SI-1. Extraction efficiency calculation

DNA (pg) = genome size (bp) / (0.978×10^9) (pg/bp)

Genome size (bp)	
E. coli	4,585,620
M. abscessus	5,090,491

(A) Calculated DNA content of one bacterial cell		
E. coli	0.0047	pg
M. abscessus	0.0052	pg

(B) From DAPI			Theoretical DNA yield (A x B)	
			pg	ng
E. coli	1.64E+07	cells/L	76895.88	76.90
M. abscessus	1.36E+07	cells/L	70788.01	70.79

Average extracted DNA (ng/µl)		Standard deviation (ng/µl)	Average total extracted DNA (ng) from 100 µl elution	Standard deviation (ng) from 100 µl elution
E. coli	0.60	0.08	60.40	7.72
M. abscessus	0.19	0.04	18.90	4.40

Average extraction efficiency		Standard deviation
E. coli	78.55%	9.53%
M. abscessus	26.70%	6.34%
