# Supplementary material for: A High-Throughput Approach for Identification of Nontuberculous Mycobacteria in Drinking Water Reveals Relationship between Water Age and Mycobacterium avium
Source: mBio. 2018 Feb 13;9(1):e02354-17. doi: 10.1128/mBio.02354-17 (PMC5821076; doi:10.1128/mBio.02354-17)
Supplement: TEXT S2 [file mbo001183725s2.pdf]

SI-2. *rpoB* and PacBio primers and thermocycling conditions

Use	Primer Name	Sequence (5' to 3')
Forward Degenerate *rpoB* Primer		TCCGA**Y**GAGGTGCT**S**GC**N**GA
Reverse Degenerate *rpoB* Primer		**SG**A**Y**TTGATGGTCA**R**CAG**Y**TCC
PacBio round 1 Forward Primer	myco_rpoB_2572_M13_f4	TGTAAAACGACGGCCAGTTCCGACGAGGTGCTCGCTGA
PacBio round 1 Forward Primer	myco_rpoB_2572_M13_f6	TGTAAAACGACGGCCAGTTCCGACGAGGTGCTGGCCGA
PacBio round 1 Forward Primer	myco_rpoB_2572_M13_f8	TGTAAAACGACGGCCAGTTCCGACGAGGTGCTGGCTGA
PacBio round 1 Forward Primer	myco_rpoB_2572_M13_f10	TGTAAAACGACGGCCAGTTCCGATGAGGTGCTCGCCGA
PacBio round 1 Forward Primer	myco_rpoB_2572_M13_f13	TGTAAAACGACGGCCAGTTCCGATGAGGTGCTGGCAGA
PacBio round 1 Forward Primer	myco_rpoB_2572_M13_f14	TGTAAAACGACGGCCAGTTCCGATGAGGTGCTGGCCGA
PacBio round 1 Reverse Primer	myco_rpoB_3864_M13_r1	CAGGAAACAGCTATGACCGGACTTGATGGTCAACAGCTCC
PacBio round 1 Reverse Primer	myco_rpoB_3864_M13_r3	CAGGAAACAGCTATGACCGGACTTGATGGTCAGCAGCTCC
PacBio round 1 Reverse Primer	myco_rpoB_3864_M13_r5	CAGGAAACAGCTATGACCGGATTTGATGGTCAACAGCTCC
PacBio round 1 Reverse Primer	myco_rpoB_3864_M13_r9	CAGGAAACAGCTATGACCCGACTTGATGGTCAACAGCTCC
**Round 2 Primers - Universal**		
PacBio round 2 Forward Primer	Forward_Bar01_M13	TCAGACGATGCGTCATTGTAAAACGACGGCCAGT
PacBio round 2 Forward Primer	Forward_Bar02_M13	CTATACATGACTCTGCTGTAAAACGACGGCCAGT
PacBio round 2 Forward Primer	Forward_Bar03_M13	CGTCTATATACGTATATGTAAAACGACGGCCAGT
PacBio round 2 Forward Primer	Forward_Bar04_M13	TGTGTATCAGTACATGTGTAAAACGACGGCCAGT
PacBio round 2 Forward Primer	Forward_Bar05_M13	ACACGCATGACACACTTGTAAAACGACGGCCAGT
PacBio round 2 Forward Primer	Forward_Bar06_M13	GATCTCTACTATATGCTGTAAAACGACGGCCAGT
PacBio round 2 Forward Primer	Forward_Bar07_M13	GCGTATATCTCATGCGTGTAAAACGACGGCCAGT
PacBio round 2 Forward Primer	Forward_Bar08_M13	ATGATGTGCTACATCTTGTAAAACGACGGCCAGT
PacBio round 2 Reverse Primer	Reverse_Bar01_M13	CTATAGACACAGCGATCAGGAAACAGCTATGACC

SI-2. *rpoB* and PacBio primers and thermocycling conditions

PacBio round 2 Reverse Primer	Reverse_Bar02_M13	CACGCTATCAGTGAGACAGGAAACAGCTATGACC
PacBio round 2 Reverse Primer	Reverse_Bar03_M13	TACATGATAGACGACACAGGAAACAGCTATGACC
PacBio round 2 Reverse Primer	Reverse_Bar04_M13	GATGAGATCTCGTGTGCAGGAAACAGCTATGACC
PacBio round 2 Reverse Primer	Reverse_Bar05_M13	AGTATCATGTGTATCTCAGGAAACAGCTATGACC
PacBio round 2 Reverse Primer	Reverse_Bar06_M13	GCACGTCGTATATCGACAGGAAACAGCTATGACC
PacBio round 2 Reverse Primer	Reverse_Bar07_M13	ATATGTGCAGTCGAGACAGGAAACAGCTATGACC

Bold and underlined bases in the original degenerate primers represent the changes made from the original Macheras *et al.* (2011) primers.
Blue text is the part of the primer that amplifies the *rpoB* gene.
16 bp PacBio barcodes are presented in red.

PCR THERMOCYCLING CONDITIONS

PCR1

98°C x 10 s
98°C x 1 s
54°C x 5 s
72°C x 14 s
} 2 cycles
98°C x 1 s
72°C x 5 s
72°C x 14 s
} 22 cycles
72°C x 1min
4°C x forever

PCR2

98°C x 10 s
98°C x 1 s
52°C x 5 s
72°C x 14 s
} 1 cycle
98°C x 1 s
72°C x 5 s
72°C x14 s
} 24 cycles
72°C x 1 min
4°C x forever
