# Supplementary material for: A High-Throughput Approach for Identification of Nontuberculous Mycobacteria in Drinking Water Reveals Relationship between Water Age and Mycobacterium avium
Source: mBio. 2018 Feb 13;9(1):e02354-17. doi: 10.1128/mBio.02354-17 (PMC5821076; doi:10.1128/mBio.02354-17)
Supplement: TEXT S3 [file mbo001183725s3.pdf]

SI-3. Combined qPCR and PacBio data

Sample ID	Stagnation time (h)	Residence time in DS (h)	Combined water age (h)	Free chlorine (mg / L)	Total chlorine (mg / L)	Monochloramine (mg / L)	Ct (mg min / L)	Number of NTM OTUs	Total NTM (*atpE* copies/L)	Concentration of NTM based on combining qPCR and PacBio results (/ L)															
										M. abscessus	*M. avium subsp. avium*	*M. avium subsp. paratuberculosis*	*M. chelonae* strain 1	*M. chelonae* strain 2	*M. chelonae* strain 3	*M. gordonia*	*M. hominissuis*	*M. indicus pranii*	*M. intracellulare*	*M. mucogenicum* strain 1	*M. mucogenicum* strain 2	*M. phocaicum*	M. sp. YC-RL4	M. sp. QIA-37	Unidentified mycobacteria
PP8	7.50	11.50	19.00	0.14	1.60	1.46	1667.82	7	6.17E+06	3.01E+04	2.47E+05	0.00E+00	0.00E+00	3.25E+06	0.00E+00	0.00E+00	0.00E+00	1.00E+04	0.00E+00	0.00E+00	2.50E+06	0.00E+00	0.00E+00	4.84E+04	7.69E+04
PP11	7.00	12.50	19.50	0.11	2.20	2.10	2451.15	7	7.41E+06	0.00E+00	2.81E+06	6.71E+04	0.00E+00	4.08E+06	0.00E+00	0.00E+00	0.00E+00	0.00E+00	0.00E+00	3.27E+05	0.00E+00	0.00E+00	2.01E+04	1.34E+04	8.50E+04
PP14	10.00	10.50	20.50	0.04	1.47	1.43	1761.36	10	6.61E+06	4.87E+05	1.30E+06	5.69E+04	0.00E+00	4.41E+06	2.67E+05	0.00E+00	6.68E+03	5.00E+04	1.00E+04	0.00E+00	3.34E+03	0.00E+00	0.00E+00	1.65E+04	0.00E+00
PP9	8.00	12.50	21.00	0.09	2.60	2.51	3162.60	9	7.24E+06	1.84E+04	1.15E+06	9.18E+03	0.00E+00	5.85E+06	0.00E+00	0.00E+00	0.00E+00	6.12E+03	0.00E+00	0.00E+00	3.07E+03	4.59E+04	0.00E+00	9.18E+03	1.50E+05
PP10	10.50	11.00	21.50	1.64	2.50	0.86	1110.69	8	6.92E+06	2.93E+06	9.27E+05	1.02E+04	0.00E+00	2.93E+06	0.00E+00	0.00E+00	0.00E+00	0.00E+00	0.00E+00	7.75E+04	0.00E+00	0.00E+00	3.43E+04	2.55E+03	2.55E+03
PP12	6.50	19.50	26.50	0.08	2.10	2.03	3219.75	9	6.17E+06	1.41E+06	1.43E+06	8.47E+04	0.00E+00	2.82E+06	0.00E+00	7.41E+04	5.83E+04	0.00E+00	0.00E+00	0.00E+00	1.27E+05	7.41E+04	0.00E+00	9.00E+04	0.00E+00
PP15	6.50	21.00	27.50	1.39	2.30	0.91	1494.90	7	5.25E+06	6.17E+05	7.08E+05	8.41E+03	0.00E+00	3.26E+06	6.12E+05	0.00E+00	0.00E+00	0.00E+00	0.00E+00	0.00E+00	0.00E+00	0.00E+00	0.00E+00	1.35E+04	2.69E+04
PP3	6.00	25.00	31.00	0.98	2.00	1.03	1906.50	8	8.34E+06	1.41E+05	7.24E+06	9.92E+04	0.00E+00	5.07E+04	0.00E+00	0.00E+00	0.00E+00	3.67E+05	0.00E+00	0.00E+00	0.00E+00	3.91E+05	0.00E+00	8.45E+03	4.01E+04
PP4	6.50	25.00	31.50	0.18	1.90	1.72	3245.13	8	8.54E+06	0.00E+00	7.57E+06	7.63E+04	0.00E+00	4.85E+04	0.00E+00	3.33E+05	0.00E+00	1.66E+05	0.00E+00	0.00E+00	0.00E+00	2.36E+05	0.00E+00	1.62E+04	9.25E+04
PP13	6.00	26.00	32.00	0.04	2.30	2.26	4339.20	9	5.37E+06	3.96E+03	5.28E+06	3.96E+03	2.38E+04	1.98E+04	0.00E+00	0.00E+00	3.96E+03	0.00E+00	0.00E+00	0.00E+00	1.79E+04	3.96E+03	0.00E+00	0.00E+00	9.92E+03
PP2	6.00	38.00	44.00	0.00	0.50	0.50	1320.00	4	7.08E+06	4.22E+05	6.51E+06	2.27E+04	0.00E+00	0.00E+00	0.00E+00	0.00E+00	0.00E+00	0.00E+00	0.00E+00	0.00E+00	0.00E+00	0.00E+00	0.00E+00	0.00E+00	1.21E+05
PP7	6.00	42.00	48.00	0.16	2.70	2.54	7315.20	6	6.31E+06	3.74E+04	5.54E+06	3.75E+03	0.00E+00	6.92E+05	0.00E+00	0.00E+00	0.00E+00	0.00E+00	0.00E+00	0.00E+00	0.00E+00	0.00E+00	0.00E+00	5.61E+03	2.81E+04
PP1	6.00	54.00	60.00	1.26	2.90	1.64	5896.80	4	5.75E+06	0.00E+00	5.48E+06	4.00E+04	0.00E+00	0.00E+00	0.00E+00	0.00E+00	0.00E+00	2.23E+05	0.00E+00	0.00E+00	0.00E+00	0.00E+00	0.00E+00	4.03E+03	0.00E+00
PP6	6.50	102.00	108.50	0.42	2.20	1.78	11613.84	7	7.24E+06	0.00E+00	7.08E+06	1.81E+04	0.00E+00	2.21E+04	0.00E+00	0.00E+00	0.00E+00	5.22E+04	0.00E+00	0.00E+00	0.00E+00	5.42E+04	0.00E+00	4.02E+03	8.03E+03
PP5	13.00	113.00	126.00	0.89	1.30	0.41	3069.36	5	9.11E+06	0.00E+00	8.91E+06	2.88E+04	0.00E+00	0.00E+00	3.77E+04	0.00E+00	0.00E+00	0.00E+00	1.11E+04	0.00E+00	0.00E+00	0.00E+00	0.00E+00	0.00E+00	1.15E+05
