# Supplementary material for: A High-Throughput Approach for Identification of Nontuberculous Mycobacteria in Drinking Water Reveals Relationship between Water Age and Mycobacterium avium
Source: mBio. 2018 Feb 13;9(1):e02354-17. doi: 10.1128/mBio.02354-17 (PMC5821076; doi:10.1128/mBio.02354-17)
Supplement: TEXT S4 [file mbo001183725s4.pdf]

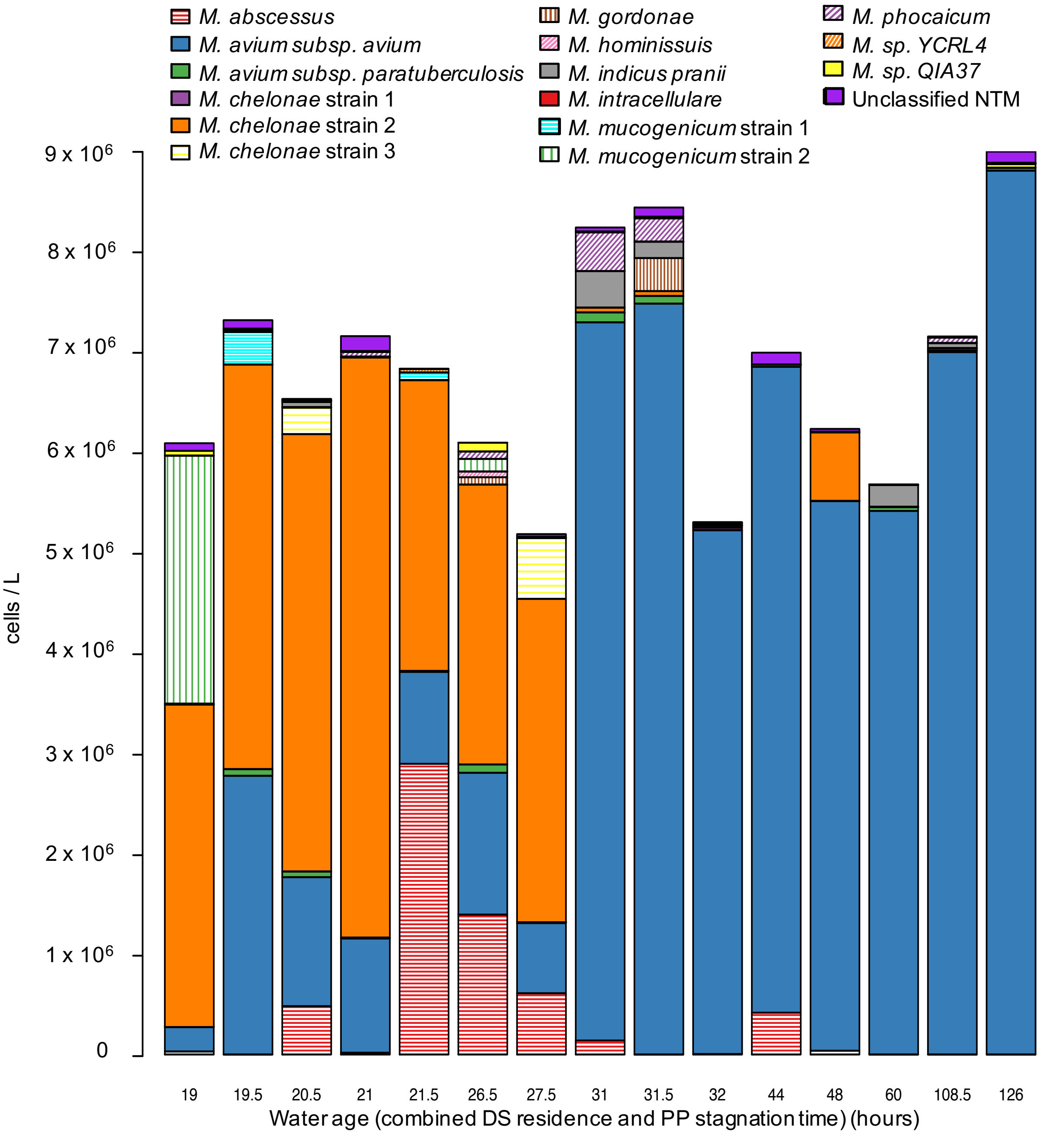

SI-4. Concentration (cells/L) of NTM species and strains in biomass from 15 drinking water samples collected from premise plumbing. The concentrations were calculated by combining qPCR and PacBio data. Water age represents the total of water residence time in the distribution system and stagnation time in premise plumbing.
