# Supplementary material for: A High-Throughput Approach for Identification of Nontuberculous Mycobacteria in Drinking Water Reveals Relationship between Water Age and Mycobacterium avium
Source: mBio. 2018 Feb 13;9(1):e02354-17. doi: 10.1128/mBio.02354-17 (PMC5821076; doi:10.1128/mBio.02354-17)
Supplement: TEXT S5 [file mbo001183725s5.pdf]

SI-5. DNA extraction procedure

Lysis Buffer – *Modified from Hill et al., Universal Nucleic Acid Extraction lysis buffer (UNEX)*

Using pre-baked glassware to remove nucleases for making Buffer A

BUFFER A

Final concentrations in buffer

2.25 M Guanidinium thiocyanate (GITC) in Tris (10 mM)- EDTA (1 mM) TE buffer (pH 8)
0.14 M Sodium Acetate
0.24 M Sodium chloride
0.4 % Sodium Sulphite
0.2 % Dithioerythritol (DTE)

Steps

- Make 5 % DTE solution
- Make 10 % sodium sulphite solution

1. Make 100 ml TE buffer solution
2. Add and dissolve 66 g of GITC into the buffer
   a. Dissolve by heating in a water bath at 50 °C for 30 min, swirl every 5 min
3. Add 12 ml of 5 M sodium chloride
4. Add 12 ml of 3 M sodium acetate
5. Add 10 ml of 5 % DTE
6. Add 10 ml of sodium sulphite

CELL LYSIS PROCEDURE FOR EIGHT EXTRACTIONS

1. Take the previously frozen 0.2 μM membrane which the water sample was filtered through out of the freezer to thaw slightly. These should be in 2 ml screw-cap tubes.
2. Heat 5123.7 μl of Buffer A in a water bath at 65 °C (warm for at least 10 min); heating the buffer to 65 °C is necessary to ensure that the SDS and Tween20 do not precipitate.
3. Add 500 μl of phenol-chloroform-isoamyl (25:24:1) pH 8 to each 2 ml screw-cap tube containing filtered biomass and vortex
4. Remove Buffer A from the water bath and
   a. Add 25.6 μl 20 % SDS
   b. Add 2 μl 20 % Tween20
   c. Add 48.7 μl proteinase K (12.9667 U/ml/min)
5. Add 600 μl of Buffer A containing the SDS, Tween20 and Proteinase K to each 2 ml screw-cap tube from step 3.
6. Vortex briefly and then incubate at 65 °C for 15 min
7. Add 0.5 g pre-baked zirconium bead (0.1 mm) and vortex
8. Bead-beat for 5 min

SI-5. DNA extraction procedure

9. Centrifuge at 13,000 g for 5 min
10. Transfer the aqueous phase to a new microcentrifuge tube
11. Add 500 µl of chloroform-isoamyl alcohol (24:1)
12. Mix by inverting
13. Centrifuge at 13,000 g for 5 min
14. Transfer aqueous phase to a new microcentrifuge tube
15. Precipitate DNA by adding 300 µl of 7.5M ammonium acetate (assuming your lysate is 600 µl, as a final concentration of 2.5M is needed)
16. Add 600 µl of isopropanol (assuming your lysate is 600 µl)
17. Add 3.3 µl of Glycoblue (15mg/ml)
18. Incubate at -20 °C overnight
19. Centrifuge for 30 min at 13,000 g
20. Remove supernatant (DNA should be pelleted to the back wall)
21. Add 500 µl of 80 % ethanol (v/v) to wash the pellet
22. Centrifuge at 13,000 g for 30 min
23. Remove ethanol and let the tube to air-dry
24. Resuspend the pellet in 30 – 50 µl of DNA-free water
25. For best results leave the pellet to dissolve into the water by leaving it at 4 °C overnight
