# Supplementary material for: A High-Throughput Approach for Identification of Nontuberculous Mycobacteria in Drinking Water Reveals Relationship between Water Age and Mycobacterium avium
Source: mBio. 2018 Feb 13;9(1):e02354-17. doi: 10.1128/mBio.02354-17 (PMC5821076; doi:10.1128/mBio.02354-17)
Supplement: TEXT S6 [file mbo001183725s6.pdf]

SI-6: qPCR primers and thermocycling conditions

Target	Forward (5'-3')	Reverse (5'-3')	Amplicon size	Annealing temp (°C)	Ref.
Mycobacteria (*atpE* gene)	FatpE: CGGYGCCGGTATCGGYGA	RatpE: CGAAGACGAACARSGCCAT	182 bp	59.6	1
Total bacteria (16S rRNA gene)	Eub338: ACTCCTACGGGAGGCAG	Eub518: ATTACCGCGGCTGCTGG	200 bp	54	2
Pseudomonas (16S rRNA gene)	PA-GS-F: GACGGGTGAGTAATGCCTA	PA-GS-R: CACTGGTGTTCCTTCCTATA	618 bp	56	3

[1] Radomski N, Roguet A, Lucas FS, Veyrier FJ, Cambau E, Accrombessi H, Moilleron R, Behr MA, Moulin L. 2013. *atpE* gene as a new useful specific molecular target to quantify Mycobacterium in environmental samples, BMC Microbiol 13:277-290.

[2] Haig SJ, Quince C, Davies RL Dorea CC and Collins G. 2014. Replicating the microbial community and water quality performance of full-scale slow sand filters in laboratory-scale filters. Water Res 61:141–151.

[3] Spilker T, Coenye T, Vandamme P, LiPuma JJ. 2004. PCR-based assay for differentiation of *Pseudomonas aeruginosa* from other *Pseudomonas* species recovered from cystic fibrosis patients. J Clin Microbiol 42:2074–2079.
